# Crop Expansion and Conservation Priorities in Tropical Countries

**DOI:** 10.1371/journal.pone.0051759

**Published:** 2013-01-09

**Authors:** Ben Phalan, Monika Bertzky, Stuart H. M. Butchart, Paul F. Donald, Jörn P. W. Scharlemann, Alison J. Stattersfield, Andrew Balmford

**Affiliations:** 1 Department of Zoology, University of Cambridge, Cambridge, United Kingdom; 2 United Nations Environment Programme World Conservation Monitoring Centre, Cambridge, United Kingdom; 3 BirdLife International, Cambridge, United Kingdom; 4 Royal Society for the Protection of Birds, Sandy, United Kingdom; 5 School of Life Sciences, University of Sussex, Brighton, United Kingdom; University of Durham, United Kingdom

## Abstract

Expansion of cropland in tropical countries is one of the principal causes of biodiversity loss, and threatens to undermine progress towards meeting the Aichi Biodiversity Targets. To understand this threat better, we analysed data on crop distribution and expansion in 128 tropical countries, assessed changes in area of the main crops and mapped overlaps between conservation priorities and cultivation potential. Rice was the single crop grown over the largest area, especially in tropical forest biomes. Cropland in tropical countries expanded by c. 48,000 km^2^ per year from 1999–2008. The countries which added the greatest area of new cropland were Nigeria, Indonesia, Ethiopia, Sudan and Brazil. Soybeans and maize are the crops which expanded most in absolute area. Other crops with large increases included rice, sorghum, oil palm, beans, sugar cane, cow peas, wheat and cassava. Areas of high cultivation potential—while bearing in mind that political and socio-economic conditions can be as influential as biophysical ones—may be vulnerable to conversion in the future. These include some priority areas for biodiversity conservation in tropical countries (e.g., Frontier Forests and High Biodiversity Wilderness Areas), which have previously been identified as having ‘low vulnerability’, in particular in central Africa and northern Australia. There are also many other smaller areas which are important for biodiversity and which have high cultivation potential (e.g., in the fringes of the Amazon basin, in the Paraguayan *Chaco*, and in the savanna woodlands of the Sahel and East Africa). We highlight the urgent need for more effective sustainability standards and policies addressing both production and consumption of tropical commodities, including robust land-use planning in agricultural frontiers, establishment of new protected areas or REDD+ projects in places agriculture has not yet reached, and reduction or elimination of incentives for land-demanding bioenergy feedstocks.

## Introduction

### Cropland expansion as a threat to biodiversity

No human activity has altered the face of the planet more than agriculture [1]–[3]. Cropland covers at least 12% of the planet's ice-free surface, and annually we now harvest more than 10% of the Earth's net primary production in the form of crops [4], [5]. Although some species can benefit from agriculture [6], habitat loss resulting from its expansion is one of the greatest global threats to biodiversity [7]–[10] and threatens to undermine progress towards meeting the Aichi Biodiversity Targets [11]. Despite this, there have been few attempts to summarise and synthesise information on global patterns of crop expansion or cultivation potential in relation to priority areas for biodiversity conservation, or to carry out systematic assessments to identify which crops might pose the greatest threat to biodiversity [10], [12], [13].

Increases in food production in recent years owe more to intensification of crop production than to cropland expansion [14]. Projections suggest that land expansion will account for only 20% of production increases in developing countries in coming decades, with higher yields (including through increased multiple cropping and shorter fallow periods) accounting for the rest [14], [15]. But despite its modest contribution to global food production, meeting 20% of production increase from new cropland by 2030 would require conversion to crop production of an area equivalent to South Africa. Most of this land is likely to be in sub-Saharan Africa and South America [16]. Although the rate of global cropland expansion is slowing, there is little room for conservationists to be complacent: new croplands have in recent decades come largely at the expense of natural habitats, particularly tropical forests [17], [18]. New markets such as those for liquid biofuels are creating new demand for agricultural products [19]. The net effects on biodiversity of increased biofuel production depends on whether biofuels ameliorate climate change impacts sufficiently to offset their land-use impacts. If even a small proportion of crop-based biofuels are planted on previously carbon-rich land, or cause indirect land use change onto such land, biofuels overall will not help to reduce greenhouse gas emissions, at least in the near term [20]. Proposals for reducing emissions from deforestation and forest degradation (REDD) for climate mitigation might help to slow cropland expansion into forests, but there is also a risk that they will displace expansion into non-forest biomes [21]. As long as agricultural expansion continues, it seems likely to remain a major driver of biodiversity loss.

It is necessary to identify those crops that have expanded most rapidly in recent years (both in absolute and relative terms) and to assess the spatial pattern of these changes—especially in tropical biomes where most species occur—if we are to understand the current and future threats they pose. Individual crops differ enormously in their biodiversity impacts, depending on how and where they are cultivated [10]. Likewise, the drivers of expansion differ among crops, depending on socioeconomic context (e.g., whether demand is for subsistence use or overseas markets) and end uses (e.g., food, animal feed or biofuels) [22]. The impacts of crop cultivation also depend on the extent to which croplands are integrated into mosaics with natural and semi-natural habitats, in which case they might cause fragmentation over a wide area but have higher biodiversity value at a local scale; or are concentrated on a smaller total area, in which case they might have lower biodiversity value locally but affect a smaller area overall [8], [23], [24].

### Aims of this study

The aim of this paper is to provide a global overview of patterns of crop expansion in relation to conservation priorities in tropical countries. Specifically, we address the following questions:

1. Which crops cover most area in tropical countries and tropical biomes?
2. In which tropical countries has most expansion occurred in recent years, and which crops were involved?
3. How are remaining areas of cultivation potential distributed across tropical countries, particularly in relation to priority areas for biodiversity conservation?

We focus on tropical countries because they support the highest concentrations of species richness and endemism for most well-studied taxonomic groups, have large projected increases in demand for food from human populations growing in size and wealth, are experiencing high rates of habitat loss, and are seen as providing the most scope for increasing global agricultural production [14], [25]–[28]. An understanding of patterns of crop expansion across tropical countries in particular will therefore be essential if increasing conflicts between biodiversity conservation and human demands for agricultural products are to be addressed.

## Methods

### Geographic scope

#### Tropical countries

We defined tropical countries as those with at least one-third of their land area between the Tropics of Cancer and Capricorn. This included 128 tropical countries (see Table S2). We used this definition rather than a wider definition incorporating all countries with any land in the tropics because pan-tropical data on changes in the area of specific crops were only available on a whole-country level; our definition thus excludes countries such as China and the United States, which have almost all of their territory outside the tropics.

#### Tropical biomes

We clipped a global map of biomes [29] to the extent of tropical countries. Biomes included in analyses were (with shortened names used on figures in parentheses): ‘tropical & subtropical moist broadleaf forests’ (moist broadleaf forests), ‘tropical & subtropical grasslands, savannas and shrubland’ (grasslands, savannas), ‘tropical & subtropical dry broadleaf forests’ (dry broadleaf forests), ‘deserts and xeric shrublands’ (drylands), ‘tropical & subtropical coniferous forests’ (coniferous forests), ‘montane grasslands and shrublands’ (montane grasslands), ‘mangroves’ (mangroves) and ‘flooded grasslands and savannas’ (flooded grasslands). We excluded from the analyses all exclusively temperate or mediterranean biomes, and also lakes, rock and ice, and tundra.

#### Priority areas for biodiversity conservation

We obtained GIS datasets of priority areas for biodiversity conservation as summarised by Brooks et al. [30] from various sources ([31]–[39] and see Table S4). We converted these, in a WGS84 geographic projection, to a 5 min×5 min (≈10 km×10 km) grid to match crop datasets, including any 5-min grid cell which overlapped the priority areas. These data were then imported into a PostgreSQL database. We used SQL queries to calculate areas of overlap using data from a matching grid on cropland extent and cultivation potential, and on the area of each grid cell calculated in an equal-area Behrmann projection.

### Data sources and limitations

To explore the impact of different crops on priority areas for biodiversity conservation across the tropics we needed data on where they are grown and expanding, but available data vary in resolution and quality. Several land cover maps show global croplands, but they often use different definitions, with often quite different results [40]–[43]. Maps which integrate satellite-derived land cover data with subnational agricultural inventory data are probably more accurate [5], and now include global maps of individual crops [44], [45]. However, time-series of such maps are not yet available, so attempts to assess change are limited to using annual data at country level [46]. We use two sorts of such data: crop data (harvested area) for changes in area of individual crops, and land data (not differentiated by crop) for changes in cropland area [46]. (See Table S4 for further details of data sources.)

Analyses based on these global data must be interpreted critically, because their quality and consistency vary [47]. Three examples serve to illustrate the need for caution when interpreting such data:

1. India does not report any harvested area for oil palm fruit in FAOSTAT [46], although it has up to 1,780 km^2^ of oil palm plantations [48]. If this was all harvested area, it would put India in the top 10 countries globally for oil palm area.
2. The crop responsible for most deforestation in Colombia, coca, is illegal and thus cannot be included in official FAO statistics [49].
3. Particularly in many African countries, crops are often intercropped on the same land [50]. The FAO provides advice for evaluating and reporting their area [51], but doing so consistently and accurately is inevitably difficult.

There are several further reasons why the sum of crop data might not equal that of land data. First, land where annual crops are harvested more than once per year from a given area is double- or triple-counted in crop data, but counted only once in land data [14]. Double- and triple-crop rice systems in Asia account for about 25% of global rice production [52]. Second, crop data exclude areas not harvested because crops were destroyed by drought, flooding or pests, or temporarily fallow, whereas land data typically include such areas [53]. Third, some countries report only fruit-bearing area for perennial crops, while others report all planted area [54]. The first discrepancy will cause crop data to overestimate true cropland area, while the second and third will lead to underestimates. Land data may thus give a more accurate picture of overall changes in cropland area.

Maps of cultivation potential [55] must also be interpreted with caution. First, climate data and projections are downscaled from a coarser grid [56]. Second, it is difficult to predict how technologies such as crop breeding will affect agricultural potential in the future: the dramatic expansion of soybeans in the southern Brazilian Amazon [57] for example, has relied upon the development of aluminium- and low-calcium-tolerant varieties [12], [58]. Third, social and political factors are important: the disastrous Mega Rice Project in Kalimantan is an example of politically-motivated cropland expansion in an area poorly suited to rice cultivation [59]. Nevertheless, while cultivation potential is not the only factor that will affect future patterns of crop expansion, and might not be the most important factor, mapping it helps to give a broad indication of the areas that might be vulnerable to conversion in the future.

To assess the possible impacts of crop expansion on biodiversity, we compared crop maps with priority areas for biodiversity conservation [30]. Brooks et al. [30] classified nine priority templates along axes of “vulnerability” and irreplaceability, defining “vulnerable” areas as those with little remaining habitat (high levels of past habitat loss). The definition is therefore retrospective, and does not provide information on vulnerability to threats in the future. Recent analysis using global land-use change projections from the IMAGE model [60] has suggested that some of the areas identified by Brooks et al. as being of “low vulnerability”—particularly High Biodiversity Wilderness Areas—might be highly vulnerable to agricultural expansion in the coming century [61].

Our analyses did not consider other forms of land use, such as livestock grazing, forestry and residential and commercial development. Conversion to cattle pasture remains the dominant driver of deforestation in Latin America, where over three-fifths of recent global humid forest conversion has occurred [62], [63]. We focus on cropland expansion because it changes habitat structure so profoundly, can be more accurately assessed by remote sensing (compared to many forms of grazing and forestry) and is so extensive (compared to urban areas).

### Cropland extent

#### Cropland extent by country: crop data

We extracted data on the harvested area of all crops for all 128 tropical countries for the years 1999–2008, the most recent for which data exist, from FAOSTAT [46]. We summed harvested areas of each of these 146 crops in each year to produce estimates of total harvested area for each crop. We also classified crops as annual or perennial [64], and summed areas of each of these two classes for each country in each year. Crops that can be grown as either annuals or perennials were classified according to [64]. For example, cassava, cotton and sugar cane were classed as annual crops (see Table S3 for scientific names of crops).

#### Cropland extent by country: land data

For each tropical country, we extracted data for 1999–2008 on the area of ‘arable land’, which corresponds to the area occupied by annual crops, and of ‘permanent crops’ (which in turn corresponds to the area occupied by perennial crops [53]). These data are reported in aggregate, without information on specific crops.

#### Maps of tropical cropland extent

We obtained maps showing the spatial distribution of cropland [5] and of individual crops [44]. Each map shows the percentage of cropland (or of specific crops) per 5-min (≈10 km) grid cell. Other similar datasets exist [45], but the maps we used were the only ones which integrated satellite and detailed subnational inventory data, and which included all of the major tropical crops. Smaller island groups, including several of high biodiversity value such as Hawai'i, the Galápagos, the Solomons, New Caledonia and Fiji, do not feature on these maps. These were included in crop and land data totals (see above), but excluded from spatial analyses.

#### Multiple cropping

As explained above, harvested areas might in some cases overestimate actual land areas used for crops harvested more than once per year from a given area. We tested whether this would change our rankings for the 12 most important crops in our dataset (defined as those in the top 10 crops by harvested area in tropical countries, and/or the top 10 by annual area increment) by calculating the minimum harvested area for each of them using information on the distribution of multiple cropping zones. We first calculated the area of each crop grown within each of nine ‘multiple cropping zones’, using crop maps from Monfreda et al. [44] (which counts double-cropped areas twice) and cropping zones from plate 13 of Fischer et al. [65]. We then divided the harvested area found in each cropping zone by the number of harvests of that crop obtainable in a year in that zone (ranging from none to three) [65]. The sum of these smaller areas gave a minimum estimate of the actual area occupied by each crop. Analyses were carried out using a Behrmann equal-area projection in ArcGIS 9.3 [66]. Crop rasters in geographic projection (WGS84) were converted to polygons, and polygon-in-polygon analyses were used to calculate the proportion of each crop in each zone.

#### Cropland extent by tropical biome

We estimated the proportion of each tropical biome occupied by cropland based on the map from [5] in ArcGIS, using similar methods to those described for multiple cropping (above). We did this in two ways. First, for each biome we calculated the mean proportion of land occupied by cropland, weighted by cell area, using information on the percentage of each 5-min grid cell occupied by cropland [5]. Second, we calculated the number and area of 5-min grid cells in each biome where there is cropland covering <10% and ≥10% of land. This second method better captures the extent of agricultural landscapes, roughly equivalent to the “villages” and “croplands” of [3], across tropical countries.

#### Crop composition by tropical biome

We estimated the proportion of each tropical biome occupied by each of the 12 most important tropical crops, using the same method as for cropland. We calculated the proportion of each biome occupied by each crop, using crop maps for the year 2000 from [44].

### Cropland expansion

#### Individual crops

To estimate the mean annual increment in harvested area of each crop across the tropics, we used linear regression of crop area on the years 1999 to 2008. We also calculated the minimum annual increment (taking account of multiple cropping) by adjusting the annual increment by our crop specific ratios of harvested area: minimum harvested area. We used regression to estimate annual change—rather than a simple comparison of area in 1999 with that in 2008—because using data points for each year (rather than just the start and end years) reduces the chance of inaccuracies in reporting having a large influence on trends, though we also looked at results based just on the difference in crop areas between 1999 and 2008.

#### Cropland expansion by country

We estimated mean annual increments for annual and perennial crops for each country, using both crop data and land data (see previous section). We used linear regression of cropland area on year to produce estimates of annual change for each country.

### Cropland potential

#### Mapping cultivation potential

To map the extent to which areas of highest cultivation potential are already occupied by cropland, we used maps of “agro-climatically attainable yield” for the 12 most important tropical crops [55]. Maps were averaged projections of yield over the period 2010 to 2030, based on a mid-range climate scenario (H3B2). We assumed an intermediate input level, except for crops mainly grown as cash crops (rice, wheat, soybeans, sugar cane and oil palm), where we assumed a high input level. For each 5-min grid cell, we could determine the potential yield for each crop, as a percentage of the tropical maximum for that crop. We took the value for the crop with the highest percentage in a grid cell as an indicator of cultivation potential, to produce combined maps of cultivation potential for crops with similar requirements for wetter climates (cassava, rice, sugar cane and oil palm) and for drier climates (beans, cow peas, groundnut, maize, millet, sorghum and soybeans), and of cultivation potential for the top 12 crops combined.

#### Cultivation potential in relation to priority areas for biodiversity conservation

We quantified the extent to which conservation priority areas in tropical countries are already occupied by cropland, and the extent to which the remaining land in these areas is suitable for rainfed crop production. We obtained shapefiles of the nine conservation priority templates presented in [30], clipped these to the extent of tropical countries, and converted them to a 5 min×5 min grid. We calculated an area-weighted mean of cropland extent within each template, using a map of cropland extent [5]. We then calculated the mean “cultivation potential” (as defined above) of the remaining land within each template, after subtracting land already converted to cropland. Grid cells for which cultivation potential was undefined, or that were located in water bodies, were not included in this calculation.

## Results

### Cropland extent

#### Crop data

**Figure 1:**
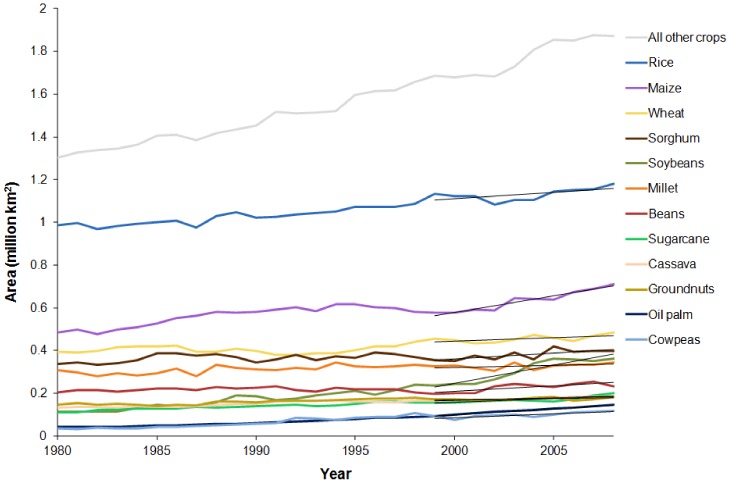
Harvested area of major crops in tropical countries, 1980–2008. The top ten crops in terms of their area in 2008 are shown. Oil palm and cow peas, which were the only two crops not on this list but which were in the top ten by area increase from 1999–2008, are also shown. Harvested areas of all other crops than these 12 are combined. Linear regressions used to assess recent rates of change in harvested area are shown. Source: [46].

The three crops with the greatest harvested area in tropical countries in 2008 (Figure 1, Table S1) were also those with the greatest harvested area globally: rice, maize and wheat [46]. The 10 most important crops by harvested area, which collectively make up two-thirds of all harvested area in tropical countries, also included sorghum, soybeans, millet, beans, sugar cane, cassava and groundnuts. All are annual crops. Rice was grown over the largest area in tropical countries (18% of tropical cropland), whereas wheat was grown over the largest area globally. When adjusted to take account of the potential for multiple cropping (minimum harvested area), the top 10 crops remained the same, although the order changed. Of the 146 crops for which data were available, 77 were annual crops and 69 were perennial crops.

#### Land data

The total area of cropland in tropical countries in 2008, calculated by summing the area of land used to grow both annual and perennial crops, was 6.7 million km^2^. This was greater than the summed harvested area of all crops in 2008: 6.4 million km^2^, suggesting that any overestimates of area introduced by multiple cropping were more than compensated for by underestimates caused by exclusion of unharvested cropland (as described in Data Sources and Limitations). To provide some context, 6.7 million km^2^ is approximately twice the land area of India, or somewhat smaller than the land area of Australia.

#### Cropland extent by tropical biome

**Figure 2:**
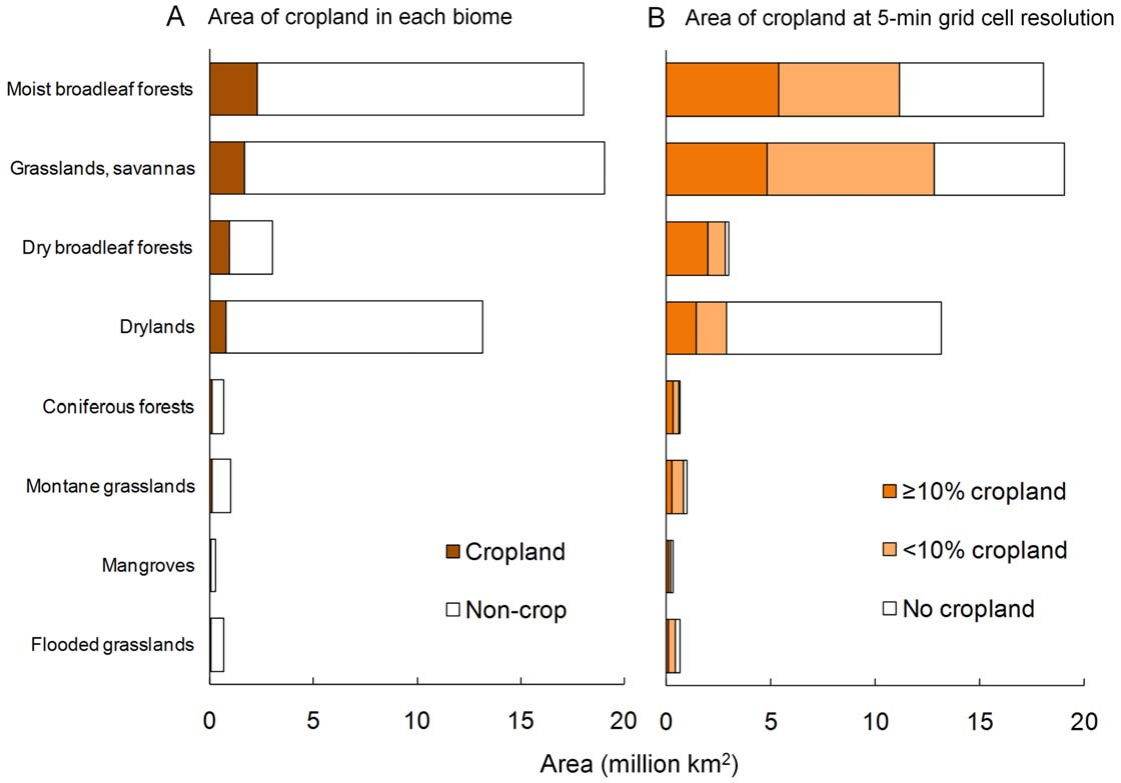
Total area of cropland in biomes within tropical countries. Shaded portions of bars show (A) total area of cropland in each biome, and (B) proportion of 5-min grid cells with <10% or ≥10% cropland cover, assessed from cropland map of [5]. Lakes, rock and ice, tundra, temperate and mediterranean biomes are excluded.

Cropland made up 10.7% of the land area of tropical countries, a little less than the global figure of 12% of ice-free land [5]. It occupied 4–17% of the area of each biome, except for dry broadleaf forests which had 32% cropland cover (Figure 2A). Summing the total area of 5-min grid cells in which there was some cropland, 62–94% of each biome had some cropland, except drylands, where the figure was 22% (Figure 2B). Counting only grid cells with at least 10% cropland, 10–67% of each biome was occupied by agricultural landscapes.

#### Crop composition by tropical biome

**Figure 3:**
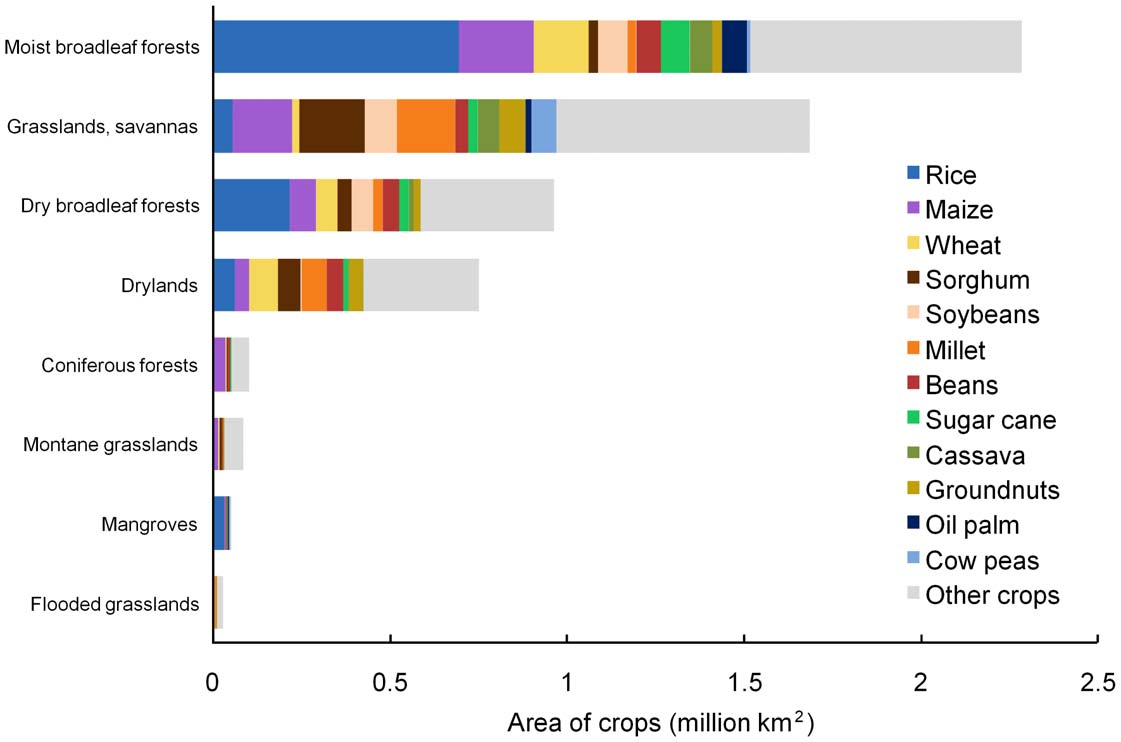
Area of different crops as a proportion of cropland in biomes within tropical countries. The top 12 tropical crops (see text) are identified. The width of each bar in this figure is equivalent to the width of the brown portions of the bars in Figure 2A. Source: [44].

Rice was the most widespread crop in the moist broadleaf forests biome, followed by maize, wheat, soybeans, sugar cane and oil palm (Figure 3). In the grassland/savanna biome, sorghum, maize and millet dominated by area. In dry broadleaf forests, rice was again most widespread, followed by maize and soybeans. In drylands, wheat and millet were most widespread. Maize dominated in the coniferous forest biome and in montane grasslands. Rice was the main crop in the mangrove biome.

### Cropland expansion

#### Overall

Across all tropical countries, cropland increased by on average ∼48,000 km^2^ per year, based on land data, or ∼98,000 km^2^ based on crop data. This equates to a rate of around 0.7% to 1.5% per year. Using only a simple change comparison between 1999 and 2008 (rather than regression models), these estimates were ∼45,000 km^2^ (land data) or ∼86,000 km^2^ (crop data) per year. As discussed in Data Sources and Limitations, the lower of each pair of estimates (based on land data) are likely to reflect more accurately land area converted to cropland, because multiple cropping and cropland from which crops were not harvested complicate reliable aggregation of area statistics from individual crops. Less than one-third of this increase (27.5%) was attributable to expansion of perennial crops (permanent crops), with the rest (72.5%) attributable to expansion of annual crops (arable land), based on regression of land data.

#### Individual crops

In terms of the mean annual area added over the period 1999–2008, soybeans and maize were by far the two most rapidly expanding crops in tropical countries (Table S1). Only one of the top 10 was a perennial crop—oil palm—which was the fifth most rapidly expanding in harvested area, or the third when adjusted for multiple cropping. The 10 most important crops by area increment, which collectively account for more than two-thirds (69.7%) of the net increase in area in tropical countries, also included rice, sorghum, beans, sugar cane, cow peas, wheat and cassava. Eight crops were shared between both top 10 lists, while millet and groundnuts featured only in the top 10 by harvested area, and oil palm and cow peas only in the top 10 by annual area increment. Results were quite similar whether a simple change comparison or regression models were used: the order changed, but the identity of the top nine crops remained the same. The simple comparison produced estimates of change which were on average 8% smaller than those from the regression models.

#### Cropland expansion by country

**Figure 4:**
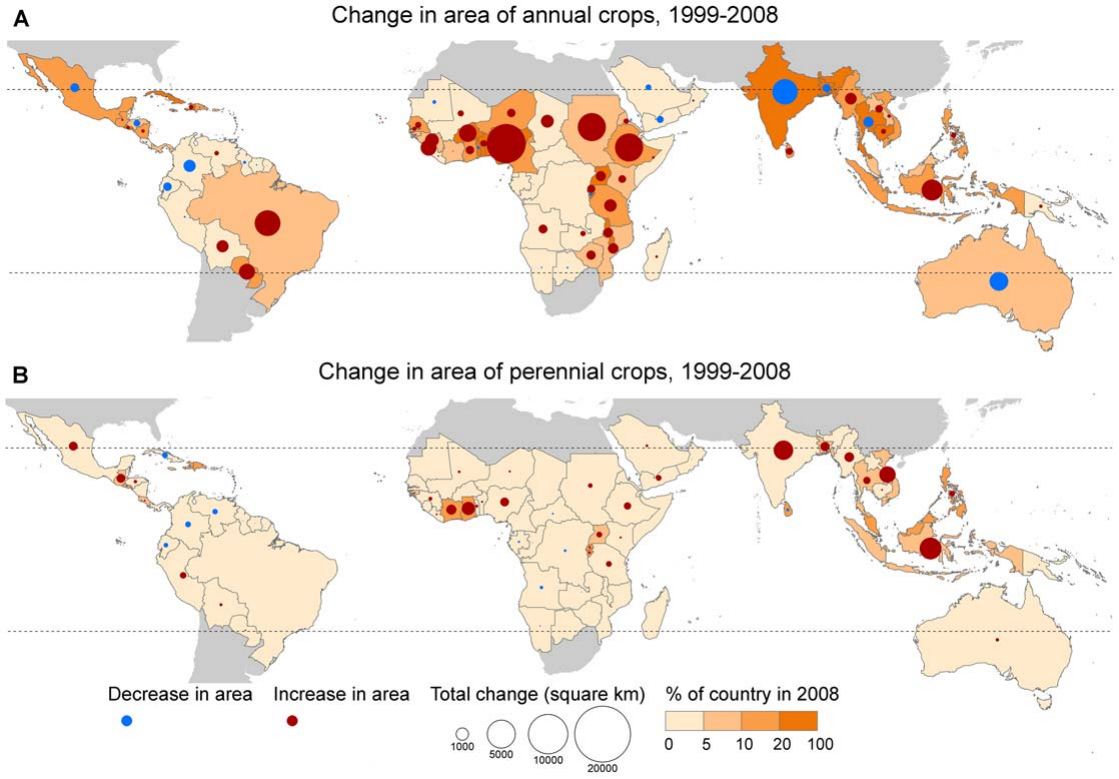
Increments in the area devoted to cropland in tropical countries. Circles show absolute increment over the period 1999–2008, with scale exaggerated 10 times for ease of interpretation. Shading indicates percentage of each country occupied by annual crops in 2008. Countries not defined as tropical are shaded grey. Maps are based on land data, for (A) arable land (annual crops) and (B) permanent cropland (perennial crops). Source: [46].

Expansion of annual crops has occurred throughout most of the tropics (red circles in Figure 4A). Based on land data, the countries which added the greatest area of annual crops (absolute increase in arable cropland) over the period 1999–2008 were Nigeria, Sudan, Ethiopia, Brazil and Indonesia. These same five countries—in a different order—also experienced the greatest increases in cropland overall. The countries in which annual crops expanded at the greatest rate (relative to the area of cropland) were Sierra Leone, Guinea, Paraguay, Ethiopia and the Gambia (see Table S2 for further details). In several countries—including India, Australia, Colombia, Mexico and Thailand—the reported area of annual crops decreased. This could have been because of cropland degradation, or a genuine contraction of annual cropland because of conversion to other uses (including perennial crops) or increases in land-use efficiency. The magnitude of changes in the area of perennial crops in tropical countries (Figure 4B) was generally smaller than that of changes in area of annual crops. While cropland area (annual and perennial crops combined) expanded in 68 of 128 tropical countries, it declined in 40 others, and remained the same in 20 countries (almost all tiny island nations).

### Cropland potential

#### Mapping cultivation potential

**Figure 5:**
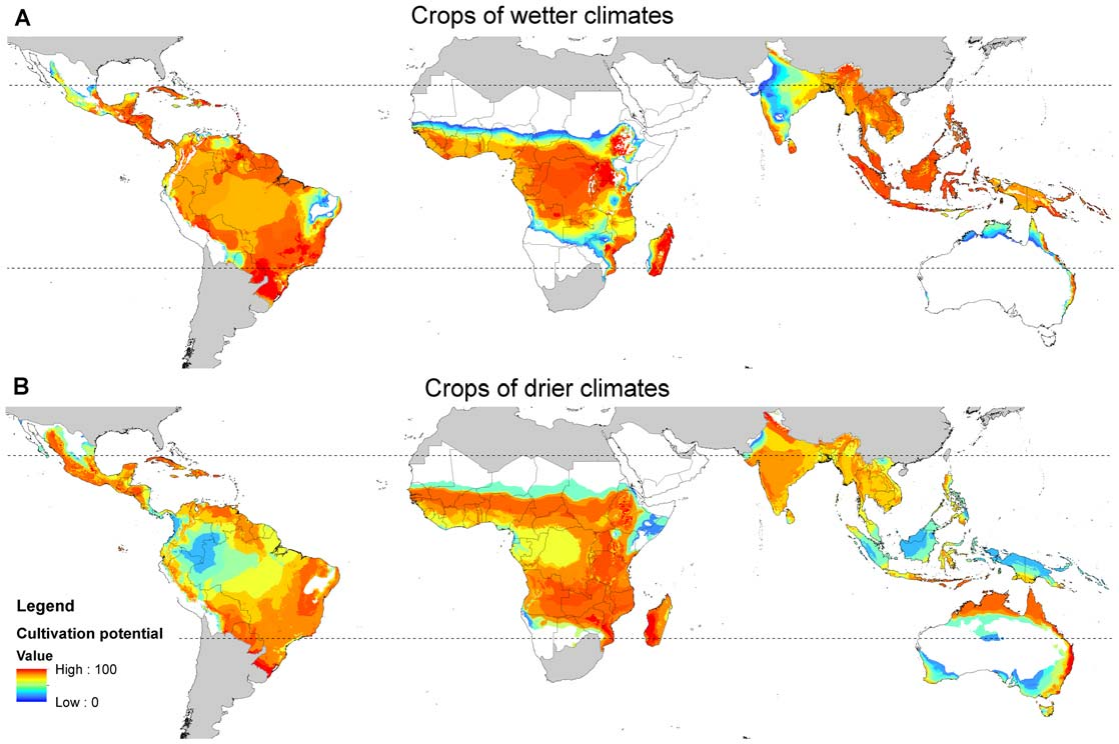
Areas of land with cultivation potential for selected crops of (A) wetter climates and (B) drier climates. Maps are based on four wetter-climate crops (cassava, rice, sugar cane and oil palm) and eight drier-climate crops (beans, cow peas, groundnut, maize, millet, sorghum, soybeans and wheat). The map shows cultivation potential for the crop for which each 5-min grid cell is most suitable. Cultivation potential is calculated as the “agro-climatically attainable yield” for each rainfed crop as a percentage of the global maximum for that crop [55].

**Figure 6:**
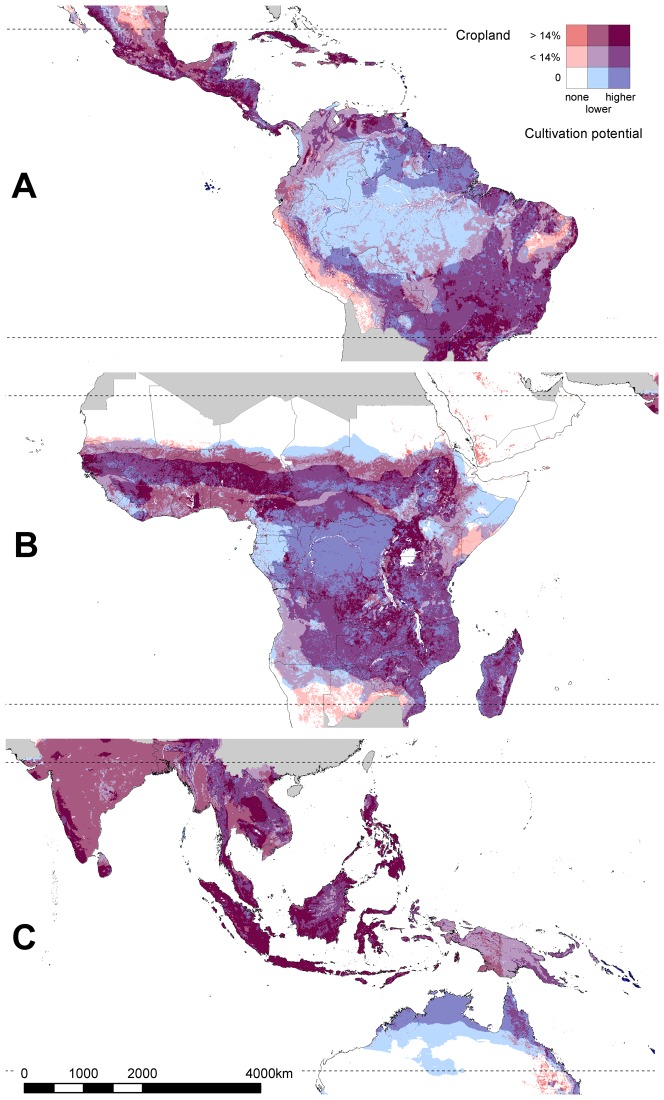
Areas of land with cultivation potential (blue) in relation to current cropland (red). This is illustrated for (A) Neotropical countries, (B) tropical Africa and (C) tropical Asia/Australia. Shades of blue indicate cultivation potential for the crop for which each 5-min grid cell is most suitable. Cultivation potential is calculated as the “agro-climatically attainable yield” for 12 major tropical crops as a percentage of the global maximum for that crop [55]. Shades of red indicate cropland extent in the year 2000, from [5]. The darker shades indicate values above the median. Land which is suitable for one or more crops, and which is already cultivated, is mapped in shades of purple. Land with no cultivation potential for these crops, and no cropland, is mapped in white, and land outside tropical countries is shaded grey.

Most land in tropical countries, with the exception of deserts and high mountains, is suitable for crop cultivation (Figure 5). There are appreciable areas believed to have cultivation potential but with little or no cropland yet (mapped in dark blue in Figure 6), particularly in the fringes of the Amazon basin, across the Congo basin, and in northern Australia. Many other parts of the tropics that are most suitable for rainfed crop production are already heavily utilised for cropland (dark purple in Figure 5). Examples include large parts of Central America, the Caribbean, south-east Brazil, large parts of the African savannas, and much of south and south-east Asia, particularly the Sundaic lowlands. In a few places, crops are grown with the aid of irrigation on land with an otherwise unsuitable climate (red in Figure 6).

#### Cultivation potential in relation to priority areas for biodiversity conservation

**Figure 7:**
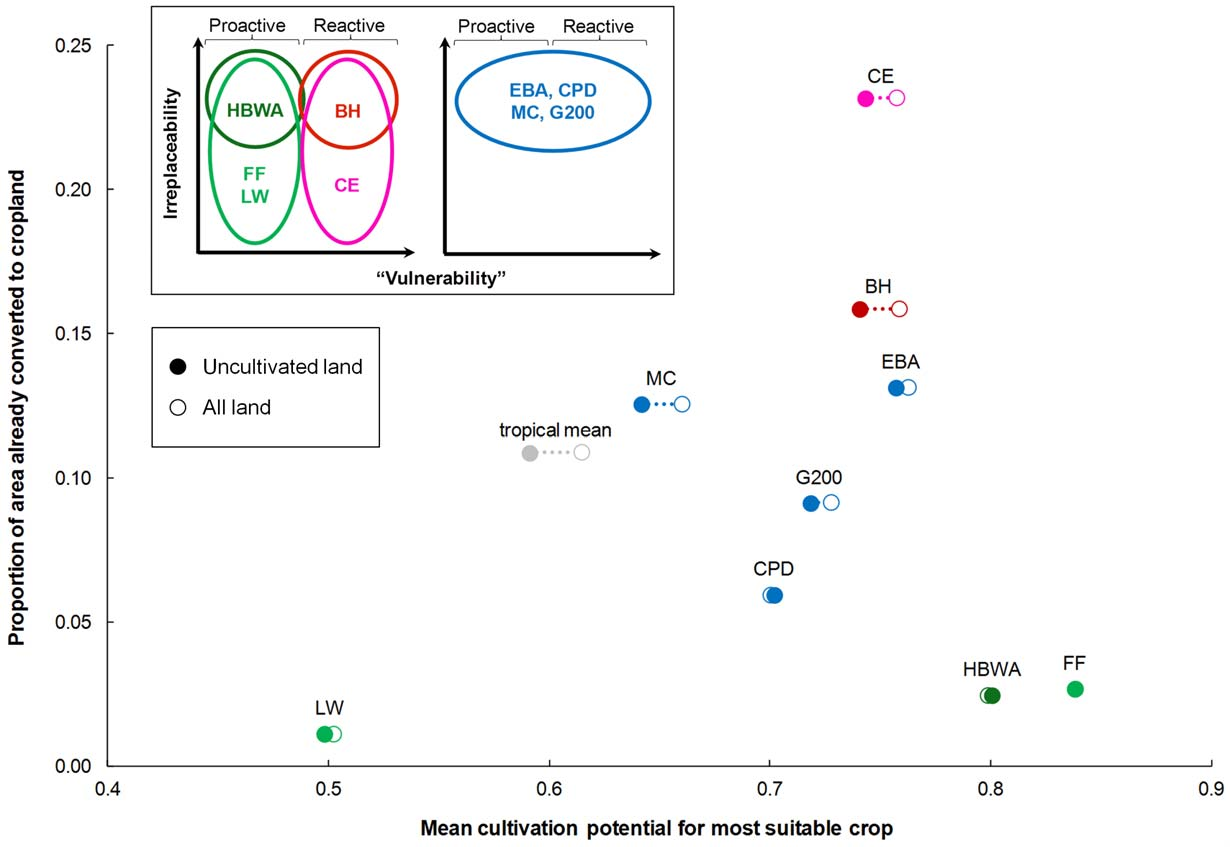
Cropland extent and cultivation potential within priority areas for biodiversity conservation in tropical countries. Cultivation potential is defined as in Figure 5. The open symbols show the mean cultivation potential of all land in each set of priority areas, while the filled symbols show the mean cultivation potential of land that had not yet been converted to cropland as of 2000. Inset from [30] shows conservation priority templates placed within the conceptual framework of irreplaceability and (retrospective) “vulnerability” and coloured accordingly (reprinted with modification, with permission from AAAS). “Proactive” conservation priorities are those in areas which are not yet considered to be highly “vulnerable” to conversion, while “reactive” priorities are those in areas where there has already been much habitat conversion. Abbreviations: Biodiversity Hotspots (BH), Centres of Plant Diversity (CPD), Crisis Ecoregions (CE), Endemic Bird Areas (EBA), Frontier Forests (FF), Global 200 Ecoregions (G200), High Biodiversity Wilderness Areas (HBWA), Last of the Wild (LW), Megadiversity Countries (MC). Mean across all tropical countries shown by grey symbols.

As would be expected, those priority templates identified by [30] as having high (retrospective) “vulnerability” (Biodiversity Hotspots and Critical Ecoregions) were also those with the largest proportion of their area already converted to cropland (Figure 7). However, there was no clear relationship between retrospective “vulnerability” and future cultivation potential. Priority areas for biodiversity conservation previously identified as having low (retrospective) “vulnerability” included those with the lowest (Last of the Wild) and highest potential for future cultivation (Frontier Forests and High Biodiversity Wilderness Areas). It would appear therefore that retrospective assessments of “vulnerability” provide little information about whether areas are biophysically suitable for conversion to cropland in the future.

## Discussion

### Cropland extent and expansion

Our analyses provide an overview of patterns of crop cultivation and expansion in tropical countries. The crops that expanded most during the period were soybeans and maize, whether or not multiple cropping is taken into account. Overall, expansion of annual crops has been more rapid and more widespread than expansion of perennial crops, and has occurred across much of South America, Africa and tropical Asia. Expansion of perennial crops—of which, oil palm has expanded most—has taken place mostly in West Africa and tropical Asia. Our analyses identified the 12 most important crops in terms of area and rate of expansion in tropical countries. Other crops which are known to be important contributors to habitat loss in specific places did not make it onto this list. Examples include cotton [67], coffee [68], tea [69], cocoa [70], rubber [71], coca [49] and pulp and paper [72].

Some of the crops which have expanded most in area in recent years are already well known drivers of biodiversity loss. Soybean expansion is recognised as a major cause of biodiversity loss in the Brazilian *Cerrado* savannas [57]. Oil palm has been described as ‘the greatest immediate threat to biodiversity in Southeast Asia’ [73]. Sugar cane has been implicated in the extinctions of species such as the Greater ‘Amakihi *Hemignathus sagittirostris* in Hawai'i and the Alagoas Curassow *Mitu mitu* (Extinct in the Wild) in Brazil [74]. Expanding maize cultivation threatens the dry forests of Madagascar [75], [76], and rice cultivation is an important cause of wetland loss [10]. Others, such as sorghum, cow peas and millet have received much less attention in the conservation literature [77], although the combined area converted to these three crops in 1999–2008 was more than twice that converted to oil palm.

There are several possible reasons why some crops have received relatively little attention from conservationists. First, area is an incomplete proxy for impact. Coffee, for example, covers a relatively small area (8% of that occupied by rice in tropical countries), but tends to replace habitats of particularly high biodiversity value. Tropical drylands support unique species, but not concentrations of endemics on the scale of tropical forests, so hectare for hectare, dryland crops might have less of an impact on biodiversity than crops of wetter climates. Second, most of the less well-known crops are traditionally grown mainly by small-scale farmers rather than on an industrial scale by large corporations. As a consequence, there is less of a clear link to Western consumers [77]. Campaigns targeting the commodity supply chains of large corporations supplying European and North American retailers have been a key factor in efforts to reduce environmental impacts of commodities such as palm oil, coffee and cocoa [78]. However, even crops traditionally seen as the preserve of subsistence farmers are increasingly grown in large-scale commercial monocultures: sorghum in parts of the Caribbean and Latin America, and cassava in Thailand and Brazil, for example [12]. Such crops are also increasingly used for biofuels and animal feed rather than to feed people [12].

### The future of wild lands

One other, very recent study has suggested that High Biodiversity Wilderness Areas might be disproportionately affected by prospective patterns of cropland expansion in coming decades, as estimated by the IMAGE model [61]. Our analysis, based more directly on maps of cultivation potential, provides further evidence that not only High Biodiversity Wilderness Areas but also Frontier Forests have the biophysical attributes that could predispose them to future conversion (indeed, all nine sets of priority areas for biodiversity conservation have considerable cultivation potential, Figure 7). High Biodiversity Wilderness Areas and Frontier Forests have been considered to have “low vulnerability” because of low levels of past habitat loss, but are likely to come under threat as infrastructure develops and if political circumstances change. The Last of the Wild priority areas have much lower cultivation potential on average because they include large areas of desert [39]. Bearing in mind that our maps are probably inaccurate at a fine spatial scale, and that biophysical cultivation potential is just one of several determinants of vulnerability to agricultural conversion, the most extensive blocks of natural habitat in areas of high cultivation potential (dark blue in Figure 6) are in central Africa, the fringes of the Amazon Basin, and northern Australia.

#### Central Africa

The extent of land with cultivation potential in the Congo Basin in central Africa is particularly alarming. Most global conservation prioritisation schemes judge this area to be at low risk [30] because of its high forest cover and low recent rates of deforestation: 0.2% to 0.4% per year [79]–[81]. However the factors that may help explain the low rates of past deforestation—such as low population densities, low road density, political instability and lack of inputs to utilise poor soils [82], [83]—are changing [84]–[87]. As a result, the “last of the wild” in central Africa is increasingly fragmented [39], and there is “not much time” [38] to protect these forests from logging followed by conversion to cassava, oil palm, rice and sugar cane. There have been reports of large-scale land acquisitions for oil palm cultivation in the Democratic Republic of Congo, including one of 28,000 km^2^ in 2007, but this appears to have been exaggerated and nothing has happened on the ground [88]. Nevertheless, the Congolian forests will continue to come under threat from expanding croplands, a threat which could be reduced by a strategic approach to road development [89], [90] and incentives for forest protection under a REDD+ mechanism [91].

#### Amazon Basin

The situation in the Amazon Basin is very different to that in central Africa. Recent deforestation has been more rapid and extensive, at least in Brazil, and thus has received far more attention from researchers and policy-makers [62], [92]. Almost half (46%) of the Brazilian Amazon has been formally protected within reserves, including indigenous reserves ([93], see Figure S1) and the rate of deforestation has declined in recent years [92]. However, legal protection for forests on private land is in danger of being weakened by changes to Brazilian legislation [94]. Our analysis confirms that most of the interior of the Amazon Basin is of relatively low suitability for agriculture, albeit still with similar cultivation potential to large parts of India or West Africa (Figure 5). Land with higher cultivation potential is concentrated around the fringes of the Amazon Basin. In unprotected areas with cultivation potential, e.g., in the Guiana Shield, creating new protected areas and setting limits on road expansion could help to reduce the threat from crop expansion [95].

#### Northern Australia

Multiple attempts to establish crops in the “empty north” of Australia have had limited success, because of intense seasonality and poor, easily eroded soils [96]. Agriculture in that area is dominated by cattle farming rather than crop production. However, minimal or no-tillage systems can enable integration of cropping and grazing, and with declining rainfall elsewhere on the continent, interest is again shifting to northern Australia. Aboriginal land and protected areas cover large parts of the suitable area mapped in Figure 6, but the risk of cropland expansion in unprotected land merits concern.

#### Other parts of the world

Other, smaller areas of high cultivation potential but as yet with little cropland are mapped in dark blue in Figure 6, and include parts of the Paraguayan *Chaco* and the savanna woodlands in the Sahel and East Africa. Areas which are suitable and already heavily farmed (dark purple) include moist and dry tropical forests in coastal Mexico and Cuba, moist tropical forests and savanna in southeastern Brazil (*Mata Atlântica* and *Cerrado*), much of West Africa (especially Nigeria), Uganda, parts of India, and much of South-east Asia. Large tracts of South America, Africa and Southeast Asia are suitable for cropland but are not yet heavily farmed (bluish purple): instead, these areas are a mosaic of croplands and fragments of semi-natural or natural habitats [3].

### Reactive or proactive conservation?

Brooks et al. [30] classify priority areas for biodiversity conservation as being either “reactive” or “proactive”. Reactive areas are those with “high vulnerability”, and include Biodiversity Hotspots and Crisis Ecoregions. Proactive areas are those with “low vulnerability”, and include High Biodiversity Wilderness Areas, Frontier Forests and the Last of the Wild. As Brooks et al. acknowledge, the measures of vulnerability that were used to identify these areas relied mainly on past patterns of habitat loss, and made no effort to be predictive. It is perhaps unsurprising, then, that we found no consistent relationship between “vulnerability” *sensu* Brooks et al., and cultivation potential (Figure 7) which is one component of vulnerability to future conversion.

What are the implications of this observation for conservation priorities? It means that much of the land which has not yet been converted to farmland has not been left alone because it is uncultivable, but because political or socio-economic factors have impeded, or at least not promoted, conversion so far. With global demand for land rising [97] areas that could previously safely be considered to have “low vulnerability” may come under increasing threat from agricultural expansion. In addition to working in areas of “high vulnerability”, conservation organisations might therefore be wise to increase their proactive conservation efforts while substantial opportunities for conservation in areas of “low vulnerability” still exist.

### Reliability of the maps and data used

The conclusions discussed above hinge on the reliability of the data used, and in particular the reliability of the maps of cultivation potential for tropical crops. As discussed in the Methods, there are good reasons to interpret these with caution. The discrepancy between our two estimates of cropland area in tropical countries is equivalent to the area of Italy, or to six years’ worth of cropland expansion. For the reasons discussed in Data Sources and Limitations, we think the larger figure of 6.7 million km^2^ is likely to be closer to the truth, but this cannot be independently verified.

In relation to the maps, the soil, terrain and climate datasets used to produce them are themselves coarse-grained and have not been comprehensively ground-truthed. Global datasets should not be taken to provide a detailed picture of conditions at finer scales. The maps do not take into account irrigation: where aquifers or rivers exist, this can transform land without sufficient rainfall for agriculture.

The maps also cannot capture new developments in agricultural technology, which allow crops to be grown where previously they could not be. A striking example is the *Cerrado* of Brazil. Until recent decades this savanna area was considered “unfit for farming” [98]. However, this is the new agricultural frontier of Latin America, where the annual area deforested for agriculture (pasture and cropland) is now on a par with that in the Amazon [99] and which is experiencing rapid expansion of cash crops such as soybeans and cotton [100], [101]. This is a clear example of how areas which are unsuitable today may become suitable in the future through developments in technology.

A key research need is therefore to reduce the uncertainties in these maps and to develop credible, fine-grained maps of cultivation potential which can be used in strategic planning, both to ensure that crops are grown where they will be most productive, and so that threats to biodiversity from agriculture can be better understood and avoided. For some crops and countries fine-grained maps have been developed (for example [102], [103]), but as they typically have not been comprehensively ground-truthed (if at all) it is difficult to know whether they are any more reliable than global datasets.

Beyond the need for better maps of cultivation potential, there is also a need for better models of future land-use change. On its own, cultivation potential is a relatively poor predictor of conversion risk. As our results here show, there are parts of the world with high cultivation potential that are not farmed, and other places with low cultivation potential which are. Other factors such as accessibility, socio-economic conditions, land tenure and government policies have as much, if not more, influence on where land conversion takes place. These drivers and policies differ greatly between countries and regions, and therefore the most promising way forward for anticipating future cropland expansion is assessments at a national or regional scale, informed by local conditions and policies (for example [104], [105]).

### Implications for policy

At the tenth Conference of the Parties to the Convention on Biological Diversity, parties agreed on 20 Aichi Biodiversity Targets [11]. These include commitments to halve, or where feasible halt, loss of natural habitats by 2020 (target 5), to ensure that areas under agriculture are managed sustainably (target 7), and to eliminate harmful pollution (target 8). The first of these can only be achieved by addressing the drivers of habitat loss, which in many parts of the world include crop expansion. To avoid compromising the first commitment, the remaining two will require yields to increase in parts of the world where productivity is currently low and where potential exists to do this without negative environmental impacts [106]. In all cases it will be difficult to reduce threats to biodiversity without strengthening public policy, such as national-level land use policies for stabilising the agricultural frontier around the last big blocks of wilderness through appropriate strategic land-use planning, infrastructure planning, better regulation of large international land acquisitions, and protected area designation.

In addition to government-led policies and incentives, there is potential for voluntary certification and other market-based initiatives to help reduce the impact of agriculture. However, this potential has been realised to only a limited extent to date. For some of the major tropical crops, including oil palm, soybeans and sugar cane, commodity roundtables have been set up to decide and implement standards for environmentally and socially responsible production [107]. These initiatives typically involve representatives from throughout the commodity chain (lenders, growers, manufacturers and retailers) as well as from governments and civil society. There is increasing attention towards the complexities and challenges of tailoring such standards to cater for small-scale farmers [108], [109]. However, these voluntary initiatives do not apply to all producers within a single country or commodity chain, and they do not have the power to implement land-use planning on the scale needed to prevent incursions of agriculture into large blocks of natural habitat, such as those in the Congo Basin. It is difficult to see how that could be achieved without government intervention, backed up by technical and financial support from wealthier governments for which tropical countries are an important source of imports and of climate-regulating ecosystem services.

All of the targets mentioned will become easier to achieve if global consumption of agricultural products can be reduced or stabilised. In the developed world, there is considerable scope to eliminate over-consumption, promote diets which are less land-demanding and reduce post-consumer waste [110], [111]. Reforming incentives for bioenergy to support only those feedstocks not implicated in direct or indirect land-use change could help to reduce global demand for agricultural land [112], [113]. In the developing world, the most important issues include rising meat consumption by an emerging middle class, rapid population growth and post-harvest losses [110], [111], [114]. Some of these issues can only be addressed by national and international policy, while others can be addressed at a local level, for example by NGOs.

Irrespective of such measures to limit over-consumption and wastage, strengthened efforts to protect wild lands from conversion will be essential if the threat of agricultural expansion to tropical biodiversity is to be reduced. Other studies have suggested that habitat conversion, once initiated in an area, is contagious and difficult to stop [115], and also that conservation in remote, less-developed parts of the world is often very cost-effective compared to conservation in more-developed areas [116]. Increasing conservation efforts in Frontier Forests and High Biodiversity Wilderness Areas may thus merit greater attention from conservationists and policy-makers.

## Supporting Information

Figure S1 **Overlap between cultivation potential and protected areas for (A) Neotropical countries, (B) tropical Africa and (C) tropical Asia/Australia.** Map of cultivation potential in relation to cropland is as for Figure 5. Protected areas comprise protected areas of all types with polygon information, extracted from the 2010 version of the World Database on Protected Areas (WDPA). [The WDPA is a joint product of IUCN and UNEP prepared by UNEP-WCMC and the IUCN-WCPA working with Governments, the Secretariats of Multilateral Environmental Agreements, collaborating Non-Government Organizations and individuals. For further information go to www.wdpa.org or contact: protectedareas@unep-wcmc.org.]
(TIF)

Table S1 **Harvested area, annual increment, % rate of expansion and regression statistics for 146 crops in tropical countries, during the period 1999–2008.** The 12 most important tropical crops (see text) are in bold.
(PDF)

Table S2 **Areas and changes in area of annual and perennial crops and total cropland, based on both crop data and land data, for the period 1999–2008, for 128 tropical countries.** Increments are based on linear regression, and all areas are in km2. Countries are ordered by annual increment in total cropland. “NA” = not available.
(PDF)

Table S3 **Scientific names of crops mentioned in the text.**
(PDF)

Table S4 **Sources of data used in this paper.** With list of references.
(PDF)
