# Supplementary figures and images for: Crop Expansion and Conservation Priorities in Tropical Countries
Source: PLoS One. 2013 Jan 9;8(1):e51759. doi: 10.1371/journal.pone.0051759 (PMC3541398; doi:10.1371/journal.pone.0051759)

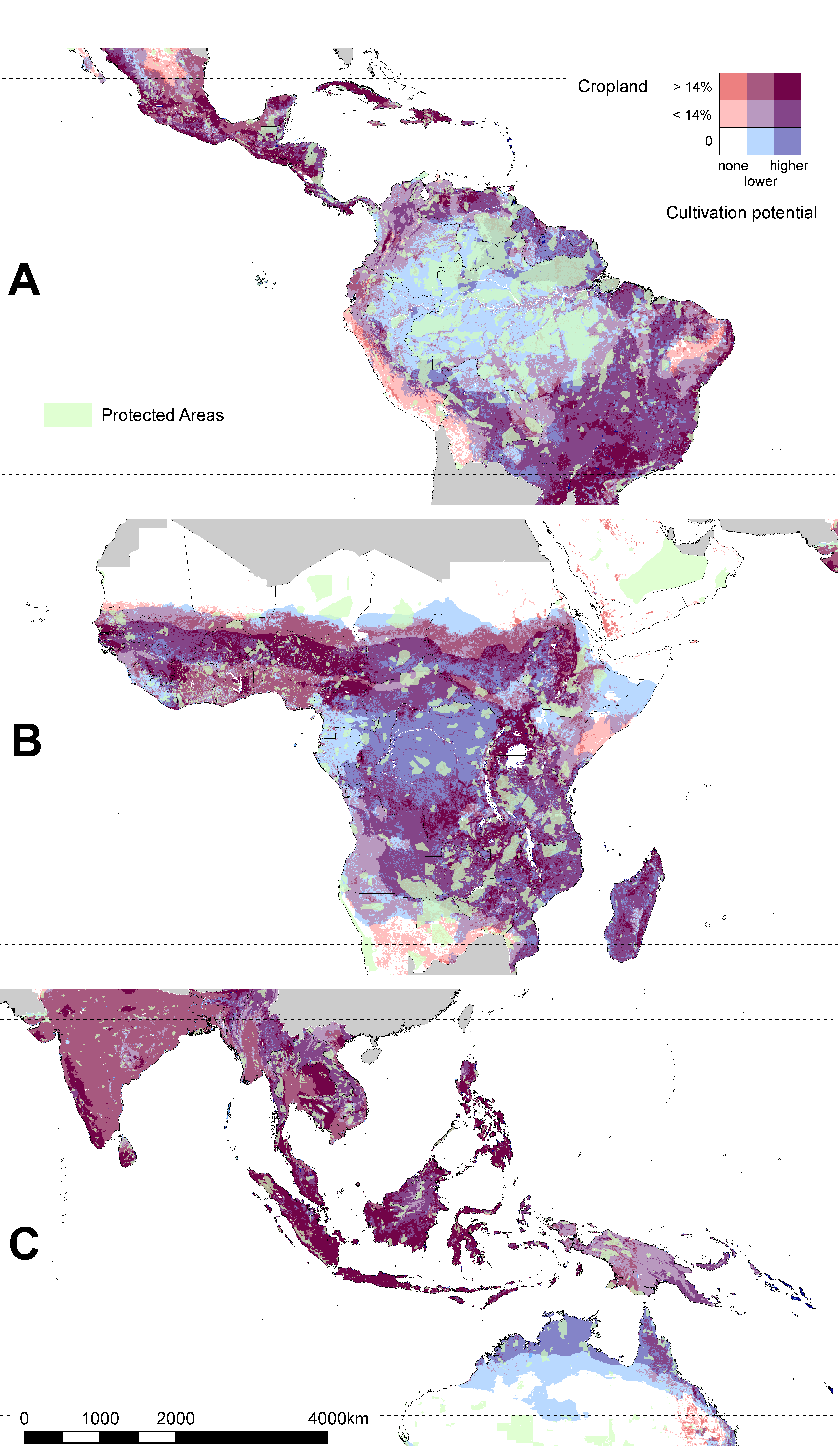

Supplement: Figure S1 — Overlap between cultivation potential and protected areas for (A) Neotropical countries, (B) tropical Africa and (C) tropical Asia/Australia. Map of cultivation potential in relation to cropland is as for Figure 5. Protected areas comprise protected areas of all types with polygon information, extracted from the 2010 version of the World Database on Protected Areas (WDPA). [The WDPA is a joint product of IUCN and UNEP prepared by UNEP-WCMC and the IUCN-WCPA working with Governments, the Secretariats of Multilateral Environmental Agreements, collaborating Non-Government Organizations and individuals. For further information go to www.wdpa.org or contact: protectedareas@unep-wcmc.org.] (TIF) [file pone.0051759.s001.tif]
