# Supplementary material for: Crop Expansion and Conservation Priorities in Tropical Countries
Source: PLoS One. 2013 Jan 9;8(1):e51759. doi: 10.1371/journal.pone.0051759 (PMC3541398; doi:10.1371/journal.pone.0051759)
Supplement: Table S1 — Harvested area, annual increment, % rate of expansion and regression statistics for 146 crops in tropical countries, during the period 1999–2008. The 12 most important tropical crops (see text) are in bold. (PDF) [file pone.0051759.s002.pdf]

Table S1. Harvested area, annual increment, % rate of expansion and regression statistics for 146 crops in tropical countries, during the period 1999–2008. The 12 most important tropical crops (see text) are in bold.

#	Crop	Harvested area 2008 (km^2)	Annual increment (km^2)	Annual rate of increase	P	R^2
1	**Soybeans**	**362,162**	**16,907**	**4.67%**	**<0.001**	**0.890**
2	**Maize**	**711,320**	**15,576**	**2.19%**	**<0.001**	**0.937**
3	**Rice, paddy**	**1,180,816**	**6,001**	**0.51%**	**0.049**	**0.402**
4	**Sorghum**	**398,759**	**5,658**	**1.42%**	**0.013**	**0.558**
5	**Oil palm fruit**	**146,016**	**5,615**	**3.85%**	**<0.001**	**0.997**
6	**Beans, dry**	**231,974**	**5,438**	**2.34%**	**0.005**	**0.653**
7	**Sugar cane**	**198,838**	**3,779**	**1.90%**	**0.002**	**0.705**
8	**Cow peas, dry**	**118,564**	**3,585**	**3.02%**	**0.002**	**0.714**
9	**Wheat**	**485,137**	**3,450**	**0.71%**	**0.048**	**0.404**
10	**Cassava**	**183,898**	**2,286**	**1.24%**	**<0.001**	**0.965**
11	Cocoa beans	86,524	2,220	2.57%	0.002	0.735
12	Barley	74,065	2,043	2.76%	0.002	0.701
13	**Millet**	**344,075**	**1,866**	**0.54%**	**0.220**	**0.181**
14	Mangoes, mangosteens, guavas	39,464	1,567	3.97%	<0.001	0.936
15	Sunflower seed	33,839	1,483	4.38%	<0.001	0.802
16	Natural rubber	83,544	1,418	1.70%	<0.001	0.873
17	Cashewnuts, with shell	41,125	1,352	3.29%	<0.001	0.956
18	Potatoes	44,933	1,285	2.86%	<0.001	0.908
19	Cereals NES	35,464	1,219	3.44%	0.028	0.472
20	Yams	49,752	1,158	2.33%	<0.001	0.985
21	Sesame seed	66,291	1,048	1.58%	0.027	0.476
22	Rapeseed	79,274	938	1.18%	0.447	0.074
23	Seed cotton	150,480	919	0.61%	0.364	0.104
24	**Groundnuts, with shell**	**183,702**	**903**	**0.49%**	**0.185**	**0.208**
25	Sweet potatoes	43,883	884	2.01%	<0.001	0.874
26	Fruit Fresh NES	28,452	844	2.97%	<0.001	0.803
27	Chick peas	86,484	809	0.94%	0.468	0.068
28	Bananas	43,759	751	1.72%	<0.001	0.903
29	Vegetables fresh NES	67,064	702	1.05%	0.228	0.176
30	Onions, dry	16,253	678	4.17%	<0.001	0.891
31	Pigeon peas	48,389	649	1.34%	<0.001	0.869
32	Fruit, tropical fresh NES	15,894	474	2.98%	<0.001	0.966
33	Plantains	53,878	472	0.88%	<0.001	0.787
34	Oats	11,496	452	3.93%	0.049	0.403
35	Arecanuts	7,507	351	4.67%	<0.001	0.951
36	Tomatoes	15,352	326	2.12%	<0.001	0.867
37	Melonseed	10,979	285	2.60%	<0.001	0.900
38	Taro (cocoyam)	15,242	285	1.87%	<0.001	0.951
39	Oranges	23,071	244	1.06%	0.012	0.562
40	Tea	13,688	237	1.73%	<0.001	0.822
41	Broad beans, horsebeans, dry	9,200	223	2.42%	0.004	0.671
42	Okra	9,367	219	2.34%	<0.001	0.898
43	Roots and Tubers NES	10,064	218	2.16%	<0.001	0.973
44	Lemons and limes	6,300	198	3.15%	0.009	0.590
45	Cauliflowers and broccoli	4,769	188	3.94%	<0.001	0.905
46	Pineapples	7,619	153	2.00%	<0.001	0.949
47	Eggplants (aubergines)	7,026	137	1.96%	<0.001	0.888
48	Ginger	3,743	130	3.48%	<0.001	0.881
49	Pepper (Piper spp.)	4,885	117	2.38%	0.010	0.583
50	Grapes	4,092	114	2.80%	<0.001	0.946

#	Crop	Harvested area 2008 (km^2)	Annual increment (km^2)	Annual rate of increase	P	R^2
51	Castor oil seed	13,045	105	0.81%	0.593	0.037
52	Coconuts	112,005	104	0.09%	0.749	0.014
53	Sisal	4,352	101	2.33%	0.001	0.740
54	Watermelons	4,302	99	2.30%	<0.001	0.903
55	Nutmeg, mace and cardamoms	2,635	99	3.74%	<0.001	0.848
56	Fonio	4,458	97	2.19%	0.007	0.621
57	Canary seed	832	92	11.11%	0.001	0.754
58	Avocados	3,028	82	2.71%	<0.001	0.977
59	Nuts NES	4,473	77	1.72%	0.014	0.552
60	Maize, green	6,370	75	1.17%	<0.001	0.899
61	Tobacco, unmanufactured	17,007	73	0.43%	0.402	0.089
62	Triticale	3,996	71	1.79%	0.094	0.311
63	Pumpkins, squash and gourds	7,748	71	0.92%	0.027	0.478
64	Cucumbers and gherkins	3,179	67	2.12%	<0.001	0.852
65	Mustard seed	929	67	7.23%	<0.001	0.936
66	Karite Nuts (Sheanuts)	4,030	66	1.64%	0.003	0.685
67	Papayas	3,984	63	1.58%	<0.001	0.909
68	Vanilla	806	60	7.50%	<0.001	0.764
69	Garlic	2,755	56	2.02%	<0.001	0.802
70	Othermelons (inc. cantaloupes)	2,014	53	2.65%	<0.001	0.871
71	Citrusfruit NES	9,640	50	0.52%	<0.001	0.940
72	Cashewapple	6,810	48	0.70%	<0.001	0.886
73	Chillies and peppers, green	7,143	45	0.63%	0.181	0.212
74	Cabbages and other brassicas	5,741	45	0.78%	0.050	0.399
75	Cinnamon (canella)	1,407	41	2.91%	<0.001	0.906
76	Oilseeds NES	11,661	40	0.35%	0.625	0.031
77	Manila Fibre (Abaca)	1,634	40	2.42%	<0.001	0.876
78	Bambara beans	1,070	38	3.52%	0.005	0.647
79	Apples	4,153	37	0.88%	0.118	0.277
80	Fibre Crops NES	2,763	31	1.13%	0.006	0.636
81	Beans, green	4,210	31	0.73%	0.019	0.517
82	Carrots and turnips	1,638	27	1.64%	<0.001	0.915
83	Spices NES	6,528	24	0.37%	0.756	0.013
84	String beans	329	23	6.91%	<0.001	0.763
85	Spinach	600	21	3.49%	<0.001	0.800
86	Walnuts, with shell	902	20	2.22%	<0.001	0.980
87	Chestnuts	403	19	4.69%	<0.001	0.853
88	Berries NES	494	18	3.55%	<0.001	0.916
89	Olives	300	17	5.58%	<0.001	0.906
90	Quinoa	785	14	1.78%	0.001	0.739
91	Dates	2,646	14	0.52%	0.009	0.594
92	Plums and sloes	512	13	2.56%	<0.001	0.868
93	Sugar crops NES	1,202	13	1.04%	<0.001	0.866
94	Yautia (cocoyam)	481	12	2.58%	0.016	0.536
95	Peas, green	4,261	11	0.26%	0.608	0.034
96	Lettuce and chicory	1,712	10	0.57%	0.004	0.667
97	Artichokes	79	10	12.26%	<0.001	0.875
98	Leguminous vegetables NES	785	10	1.22%	<0.001	0.834
99	Asparagus	485	8	1.64%	0.007	0.621
100	Anise, badian, fennel, corian	3,173	7	0.23%	0.001	0.758
101	Almonds, with shell	185	7	3.73%	0.008	0.608
102	Onions (inc. shallots), green	547	6	1.03%	0.116	0.280
103	Hops	256	5	1.79%	0.010	0.588
104	Pyrethrum, Dried	267	4	1.64%	0.026	0.481

#	Crop	Harvested area 2008 (km^2)	Annual increment (km^2)	Annual rate of increase	P	R^2
105	Apricots	127	3	2.74%	0.011	0.579
106	Persimmons	89	3	3.56%	<0.001	0.912
107	Agave Fibres NES	533	2	0.45%	0.001	0.749
108	Strawberries	145	2	1.67%	0.054	0.388
109	Maté	1,032	2	0.22%	0.796	0.009
110	Grapefruit (inc. pomelos)	1,063	2	0.20%	0.590	0.038
111	Sugar beet	33	1	3.60%	0.008	0.601
112	Buckwheat	480	1	0.24%	0.407	0.088
113	Raspberries	11	1	6.94%	<0.001	0.850
114	Chicory roots	7	1	8.79%	<0.001	0.837
115	Cherries	41	1	1.24%	<0.001	0.826
116	Stone fruit NES	43	0.4	0.90%	0.651	0.027
117	Mushrooms and truffles	8	0.3	4.35%	0.010	0.583
118	Sour cherries	5	0.2	4.67%	<0.001	0.821
119	Hazelnuts, with shell	1	0.1	12.73%	0.006	0.636
120	Ramie	35	0.1	0.30%	0.130	0.262
121	Currants	2	0.1	3.29%	0.025	0.485
122	Figs	80	0.02	0.02%	0.937	0.001
123	Carobs	1	-0.01	-2.42%	0.722	0.017
124	Pistachios	6	-0.1	-0.96%	0.132	0.260
125	Blueberries	0	-0.1	-52.21%	0.004	0.674
126	Brazilnuts, with shell	7	-0.1	-1.39%	0.549	0.047
127	Kiwi fruit	2	-0.2	-6.75%	0.070	0.354
128	Tung Nuts	166	-0.3	-0.17%	0.848	0.005
129	Peaches and nectarines	1,200	-1	-0.12%	0.788	0.010
130	Pears	424	-2	-0.43%	0.027	0.478
131	Quinces	19	-2	-9.93%	0.003	0.678
132	Rye	404	-3	-0.78%	0.047	0.409
133	Vetches	1,833	-5	-0.29%	0.770	0.011
134	Kolanuts	3,765	-9	-0.24%	0.213	0.187
135	Tangerines, mandarins, clem.	1,847	-10	-0.55%	0.011	0.579
136	Lentils	16,475	-39	-0.24%	0.743	0.014
137	Other Bastfibres	2,162	-62	-2.85%	0.002	0.719
138	Linseed	7,965	-89	-1.11%	0.274	0.147
139	Peas, dry	14,947	-90	-0.61%	0.523	0.053
140	Pulses NES	35,208	-96	-0.27%	0.632	0.030
141	Chillies and peppers, dry	16,137	-96	-0.60%	0.128	0.265
142	Jute	12,740	-100	-0.78%	0.134	0.258
143	Cloves	3,842	-128	-3.34%	0.066	0.362
144	Safflower seed	4,451	-182	-4.08%	0.013	0.556
145	Coffee, green	97,310	-488	-0.50%	0.163	0.227
146	Lupins	5,846	-778	-13.30%	<0.001	0.938
