# Supplementary material for: Crop Expansion and Conservation Priorities in Tropical Countries
Source: PLoS One. 2013 Jan 9;8(1):e51759. doi: 10.1371/journal.pone.0051759 (PMC3541398; doi:10.1371/journal.pone.0051759)
Supplement: Table S2 — Areas and changes in area of annual and perennial crops and total cropland, based on both crop data and land data, for the period 1999–2008, for 128 tropical countries. Increments are based on linear regression, and all areas are in km2. Countries are ordered by annual increment in total cropland. “NA” = not available. (PDF) [file pone.0051759.s003.pdf]

Table S2. Areas and changes in area of annual and perennial crops and total cropland, based on both crop data and land data, for the period 1999–2008, for 128 tropical countries. Increments are based on linear regression, and all areas are in km^2. Countries are ordered by annual increment in total cropland. "NA" = not available.

#	Country	Annual crops			Perennial crops			Total cropland	
		land data		crop data	land data		crop data	land data	
		2008 area	annual increment	annual increment	2008 area	annual increment	annual increment	2008 area	annual increment
1	Nigeria	375,000	9,758	6,396	30,000	500	724	405,000	10,258
2	Indonesia	220,000	2,815	442	151,000	3,011	5,100	371,000	5,826
3	Ethiopia	136,060	5,066	3,374	9,070	339	291	145,130	5,405
4	Sudan	206,980	5,086	1,879	2,080	141	8	209,060	5,227
5	Brazil	610,000	4,205	18,550	75,000	0	262	685,000	4,205
6	Burkina Faso	63,000	1,939	1,550	600	0	3	63,600	1,939
7	Vietnam	63,000	70	535	31,150	1,673	901	94,150	1,743
8	Sierra Leone	17,950	1,665	1,407	1,350	0	29	19,300	1,665
9	Paraguay	42,000	1,635	2,653	1,000	12	9	43,000	1,648
10	Ghana	44,000	465	606	28,500	1,103	935	72,500	1,568
11	Myanmar (Burma)	106,000	891	5,444	11,000	611	138	117,000	1,502
12	Guinea	24,000	1,217	924	6,900	87	86	30,900	1,304
13	Tanzania, United Republic of	96,000	962	5,258	13,500	206	357	109,500	1,168
14	Chad	43,000	1,065	902	300	0	0	43,300	1,065
15	Bolivia	36,000	905	762	2,190	62	4	38,190	967
16	Uganda	56,500	660	1,050	22,500	203	176	79,000	863
17	Mozambique	45,000	718	977	2,500	5	-37	47,500	724
18	Ivory Coast	28,000	6	462	42,500	645	1,072	70,500	652
19	Malawi	35,000	624	701	1,220	-1	-2	36,220	623
20	Zimbabwe	37,300	558	484	1,200	0	5	38,500	558
21	Guatemala	13,250	34	514	9,430	496	164	22,680	531
22	Niger	144,930	458	4,623	430	29	33	145,360	487
23	Angola	34,000	515	1,574	2,900	-67	-34	36,900	448
24	Rwanda	12,900	410	320	2,800	31	31	15,700	442
25	Kenya	53,000	373	232	5,000	31	94	58,000	404
26	Philippines	53,000	186	566	50,000	208	84	103,000	394
27	Laos	12,500	377	292	950	10	25	13,450	387
28	Benin	25,500	260	-55	2,950	35	44	28,450	295
29	Mali	48,500	237	1,266	1,300	51	59	49,800	288
30	Senegal	35,000	243	-118	540	-1	-14	35,540	242
31	Sri Lanka	12,500	298	122	9,500	-60	-94	22,000	238
32	Peru	36,500	-24	175	7,900	260	222	44,400	236
33	Zambia	23,550	199	282	290	3	-1	23,840	202
34	Cambodia	39,000	170	1,239	1,550	20	-15	40,550	190
35	Eritrea	6,700	178	191	20	-1	3	6,720	177
36	Gambia, The	3,900	124	129	50	0	0	3,950	124
37	Haiti	10,000	139	117	3,000	-19	50	13,000	120
38	Bangladesh	79,000	-441	-180	8,000	536	497	87,000	96
39	Papua New Guinea	2,700	70	45	6,500	7	36	9,200	78
40	Nicaragua	19,000	68	112	2,300	-1	36	21,300	67
41	Somalia	10,000	46	66	270	3	4	10,270	49
42	Timor-Leste	1,600	52	44	650	-3	-4	2,250	49
43	Madagascar	29,500	48	198	6,000	0	-38	35,500	48
44	El Salvador	6,850	67	-40	2,300	-23	-3	9,150	44
45	Liberia	4,000	14	64	2,180	20	38	6,180	34
46	Oman	550	29	3	390	-5	-6	940	24
47	Guinea-Bissau	3,000	10	10	2,500	14	16	5,500	23
48	Cape Verde	650	17	-14	30	1	0	680	18
49	Vanuatu	200	0	2	1,250	16	4	1,450	16
50	Solomon Islands	160	2	3	600	6	9	760	9
51	Panama	5,480	10	31	1,470	-2	3	6,950	8
52	Comoros	800	1	11	550	6	6	1,350	7
53	Sao Tome and Principe	90	4	1	450	1	-7	540	5

#	Country	Annual crops			Perennial crops			Total cropland	
		land data		crop data	land data		crop data	land data	
		2008 area	annual increment	annual increment	2008 area	annual increment	annual increment	2008 area	annual increment
54	Cameroon	59,630	4	1,581	12,000	0	120	71,630	4
55	New Caledonia	80	3	1	50	1	1	130	4
56	Dominica	50	1	0	160	3	-2	210	4
57	Western Samoa	250	-1	0	380	4	4	630	3
58	Belize	700	7	3	320	-4	-7	1,020	3
59	French Guiana	128	3	-4	43	0	0	171	3
60	French Polynesia	30	0	0	220	3	4	250	3
61	Costa Rica	2,000	-18	-23	3,000	20	32	5,000	2
62	Brunei	30	1	-5	50	1	1	80	2
63	Marshall Islands	20	2	NA	80	0	0	100	2
64	Congo	4,900	-3	49	520	3	3	5,420	0
65	Grenada	20	1	0	90	-1	-2	110	0
66	Tonga	150	0	1	120	0	0	270	0
67	Niue	10	0	0	30	0	0	40	0
68	Djibouti	10	0	-4	0	0	NA	10	0
69	American Samoa	20	0	2	30	0	0	50	0
70	Antigua and Barbuda	80	0	0	10	0	0	90	0
71	Aruba	20	0	NA	NA	0	NA	20	0
72	Barbados	160	0	-2	10	0	0	170	0
73	British Virgin Islands	10	0	NA	10	0	0	20	0
74	Cayman Islands	2	0	0	5	0	0	7	0
75	Fiji	1,700	0	0	830	0	6	2,530	0
76	Kiribati	20	0	0	320	0	5	340	0
77	Mayotte	70	0	NA	130	0	NA	200	0
78	Federated States of Micronesia	25	0	0	170	0	0	195	0
79	Montserrat	20	0	0	0	0	0	20	0
80	Nauru	0	0	0	4	0	0	4	0
81	Netherlands Antilles	80	0	NA	NA	0	NA	80	0
82	Northern Mariana Islands	10	0	NA	10	0	NA	20	0
83	Palau	10	0	NA	20	0	NA	30	0
84	Saint Helena	40	0	NA	NA	0	NA	40	0
85	Togo	24,600	-64	82	1,700	64	102	26,300	0
86	Tokelau	0	0	0	6	0	0	6	0
87	Turks and Caicos Islands	10	0	NA	NA	0	NA	10	0
88	Wallis and Futuna	10	0	0	50	0	0	60	0
89	Tuvalu	0	0	0	18	0	0	18	0
90	St. Vincent and the Grenadines	50	0	1	30	-1	2	80	-1
91	Singapore	5	-1	1	2	0	0	7	-1
92	Guam	10	-1	0	100	0	1	110	-1
93	Seychelles	10	0	0	30	-1	-3	40	-1
94	United States Virgin Islands	10	-1	0	10	0	NA	20	-1
95	Guadeloupe	211	3	-1	34	-4	-4	245	-1
96	Maldives	40	0	0	40	-2	-4	80	-2
97	Puerto Rico	600	3	-8	370	-7	-16	970	-4
98	Cook Islands	20	-2	0	10	-3	-2	30	-5
99	St. Lucia	30	1	-1	70	-6	-6	100	-5
100	St. Kitts and Nevis	40	-4	-2	1	-1	0	41	-5
101	Reunion	328	-5	-1	32	-1	1	360	-6
102	Martinique	110	0	-1	60	-6	13	170	-6
103	Namibia	8,000	-17	30	80	5	6	8,080	-12
104	Dominican Republic	8,000	-12	-64	5,000	0	46	13,000	-12
105	Mauritius	870	-14	-13	40	0	2	910	-14
106	Suriname	490	-11	-5	70	-3	0	560	-15
107	Trinidad and Tobago	250	-12	-13	220	-3	5	470	-16
108	Mexico	248,000	-524	-570	27,000	508	220	275,000	-17
109	Central African Republic	19,300	1	181	800	-19	-28	20,100	-18
110	Malaysia	18,000	-19	-52	57,850	0	898	75,850	-19

#	Country	Annual crops			Perennial crops			Total cropland	
		land data		crop data	land data		crop data	land data	
		2008 area	annual increment	annual increment	2008 area	annual increment	annual increment	2008 area	annual increment
111	Jamaica	1,250	-21	-19	1,100	0	11	2,350	-21
112	Botswana	2,500	-24	-56	20	1	0	2,520	-23
113	Equatorial Guinea	1,310	1	0	750	-27	-29	2,060	-26
114	Gabon	3,250	0	8	1,500	-30	-5	4,750	-30
115	Burundi	9,000	-83	36	3,900	44	57	12,900	-39
116	Venezuela	27,000	128	599	6,500	-173	-115	33,500	-45
117	Zaire	67,000	0	-114	7,500	-48	-72	74,500	-48
118	Guyana	4,200	-60	-32	250	-2	-6	4,450	-62
119	Yemen	12,790	-309	344	3,270	226	-11	16,060	-82
120	Mauritania	4,000	-100	62	110	-2	3	4,110	-102
121	Saudi Arabia	34,460	-206	-143	2,300	51	90	36,760	-156
122	Honduras	10,180	-263	-96	4,100	72	118	14,280	-191
123	Cuba	35,700	12	-844	4,000	-224	-80	39,700	-212
124	Thailand	152,000	-705	593	36,500	342	618	188,500	-363
125	Ecuador	12,360	-469	55	12,640	-146	-149	25,000	-615
126	Colombia	18,300	-914	35	16,310	-220	231	34,610	-1,133
127	India	1,581,450	-4,083	11,628	111,750	2,392	3,241	1,693,200	-1,690
128	Australia	440,240	-2,242	1,914	3,500	77	104	443,740	-2,165
	**GRAND TOTALS**	**5,829,359**	**34,466**	**81,320**	**895,845**	**13,088**	**16,713**	**6,725,204**	**47,554**
