# Supplementary material for: Crop Expansion and Conservation Priorities in Tropical Countries
Source: PLoS One. 2013 Jan 9;8(1):e51759. doi: 10.1371/journal.pone.0051759 (PMC3541398; doi:10.1371/journal.pone.0051759)
Supplement: Table S3 — Scientific names of crops mentioned in the text. (PDF) [file pone.0051759.s004.pdf]

Table S3. Scientific names of crops mentioned in the text.

**English name**	**Scientific name**
Beans	*Phaseolus* spp.
Cassava	*Manihot esculenta*
Coca	*Erythroxylum coca, E. novogranatense*
Cocoa	*Theobroma cacao*
Coffee	*Coffea arabica, C. canephora*
Cotton	*Gossypium hirsutum*, other *Gossypium* spp.
Cow peas	*Vigna unguiculata (=V. sinensis)*
Groundnuts	*Arachis hypogaea*
Maize	*Zea mays*
Millet	*Pennisetum glaucum, Setaria italica*, other species
Oil palm	*Elaeis guineensis, E. oleifera*
Rice	*Oryza sativa, O. glaberrima*
Rubber	*Hevea brasiliensis*
Sorghum	*Sorghum bicolor*
Soybeans	*Glycine max*
Sugar cane	*Saccharum* spp.
Tea	*Camellia sinensis*
Wheat	*Triticum* spp.
