# Supplementary material for: Crop Expansion and Conservation Priorities in Tropical Countries
Source: PLoS One. 2013 Jan 9;8(1):e51759. doi: 10.1371/journal.pone.0051759 (PMC3541398; doi:10.1371/journal.pone.0051759)
Supplement: Table S4 — Sources of data used in this paper. With list of references. (PDF) [file pone.0051759.s005.pdf]

Table S4. Sources of data used in this paper.

Description	Ref.
**Global biomes**<br>GIS data from: http://www.worldwildlife.org/science/data/item6373.html	[S1]
**Cropland area** for individual crops and for arable and permanent cropland	[S2]
**Cropland extent**, on global 5-min (~10 km) grid<br>GIS data from: http://www.geog.mcgill.ca/~nramankutty/Datasets/Datasets.html	[S3]
**Individual crop extents**, on global 5-min (~10 km) grid<br>GIS data from: http://www.geog.mcgill.ca/~nramankutty/Datasets/Datasets.html	[S4]
**Multiple cropping zones**<br>GIS data from: http://www.iiasa.ac.at/Research/LUC/SAEZ/index.html	Plate 13 in [S5]
**Cultivation potential**<br>GIS data from: http://www.iiasa.ac.at/Research/LUC/GAEZv3.0/	[S6]
**Biodiversity Hotspots**<br>Shapefile from:<br>http://www.biodiversityhotspots.org/xp/Hotspots/resources/pages/maps.aspx	[S7,S8]
**Centres of Plant Diversity**<br>Shapefile kindly provided by United Nations Environment Program World Conservation Monitoring Centre and IUCN	[S9]
**Crisis Ecoregions**<br>Identified using a list provided by Jonathan Hoekstra of WWF from ecoregions shapefile from: http://www.worldwildlife.org/science/data/item6373.html	[S10]
**Endemic Bird Areas**<br>Shapefile kindly provided by BirdLife International	[S11]
**Frontier Forests**<br>Shapefile from:<br>http://www.globalforestwatch.org/english/datawarehouse/index.asp	[S12]
**Global 200 Ecoregions**<br>Shapefile from: http://www.worldwildlife.org/science/data/item6373.html	[S13,S14]
**High Biodiversity Wilderness Areas**<br>Shapefile kindly provided by the Conservation International Foundation	[S15]
**Last of the Wild**<br>Shapefile from: http://sedac.ciesin.columbia.edu/wildareas/downloads.jsp	[S16]
**Megadiversity Countries**<br>The following countries were selected from a global map of countries: Australia, Brazil, China, Colombia, Democratic Republic of the Congo, Ecuador, India, Indonesia, Madagascar, Malaysia, Mexico, Papua New Guinea, Peru, Philippines, South Africa, United States, Venezuela	[S17]

References

S1. Olson DM, Dinerstein E, Wikramanayake ED, Burgess ND, Powell GVN, et al. (2001) Terrestrial ecoregions of the world: A new map of life on Earth. BioScience 51: 933-938.

S2. FAO (2011) FAOSTAT Statistical databases. Food and Agriculture Organization of the United Nations. Available: http://faostat.fao.org/.

S3. Ramankutty N, Evan AT, Monfreda C, Foley JA (2008) Farming the planet: 1. Geographic distribution of global agricultural lands in the year 2000. Global Biogeochem. Cycles 22: GB1003, doi:10.1029/2007GB002952.

S4. Monfreda C, Ramankutty N, Foley JA (2008) Farming the planet: 2. Geographic distribution of crop areas, yields, physiological types, and net primary production in the year 2000. Global Biogeochem. Cycles 22: GB1022.

S5. Fischer G, van Velthuizen H, Shah M, Nachtergaele F (2002) Global Agro-ecological Assessment for Agriculture in the 21st Century: Methodology and Results. Laxenburg, Austria and Rome, Italy: International Institute for Applied Systems Analysis/Food and Agriculture Organization of the United Nations. 119 p.

S6. Tóth G, Kozlowski B, Prieler S, Wiberg D (2011) Global Agro-Ecological Zones (GAEZ v3.0) User's Guide. Laxenburg and Rome: IIASA and FAO. 56 p. Available: http://www.iiasa.ac.at/Research/LUC/GAEZv3.0/docs/GAEZ_User_Guide.pdf. Accessed 28 Jun 2011.

S7. Mittermeier RA, Gil PR, Hoffmann M, Pilgrim J, Brooks T, et al. (2004) Hotspots Revisited. Mexico City: Cemex. 392 p.

S8. Myers N, Mittermeier RA, Mittermeier CG, da Fonseca GAB, Kent J (2000) Biodiversity hotspots for conservation priorities. Nature 403: 853-858.

S9. WWF, IUCN (1997) Centres of Plant Diversity. Gland, Switzerland: WWF and IUCN. 3 vols.

S10. Hoekstra JM, Boucher TM, Ricketts TH, Roberts C (2005) Confronting a biome crisis: global disparities of habitat loss and protection. Ecol. Lett. 8: 23-29. doi:10.1111/j.1461-0248.2004.00686.x

S11. Stattersfield AJ, Crosby MJ, Long AJ, Wege DC (1998) Endemic Bird Areas of the World: Priorities for Biodiversity Conservation. BirdLife Conservation Series no. 7. Cambridge, UK: BirdLife International. 846 p.

S12. Bryant D, Nielsen D, Tangley L (1997) Last frontier forests: Ecosystems and economies on the edge. Washington DC, USA: World Resources Institute. 54 p.

S13. Olson DM, Dinerstein E (1998) The Global 200: a representation approach to conserving the Earth's most biologically valuable ecoregions. Conserv. Biol. 12: 502-515.

S14. Olson DM, Dinerstein E (2002) The Global 200: Priority Ecoregions for global conservation. Ann. Missouri Bot. Gard. 89: 199-224. doi:10.2307/3298564

S15. Mittermeier RA, Mittermeier CG, Brooks TM, Pilgrim JD, Konstant WR, et al. (2003) Wilderness and biodiversity conservation. Proc. Natl. Acad. Sci. U.S.A. 100: 10309 - 10313. doi:10.1073/pnas.1732458100

S16. Sanderson EW, Jaiteh M, Levy MA, Redford KH, Wannebo AV, et al. (2002) The Human Footprint and the Last of the Wild. BioScience 52: 891-904. doi:10.1641/0006-3568(2002)052[0891:THFATL]2.0.CO;2

S17. Mittermeier RA, Robles Gil P, Mittermeier CG (1997) Megadiversity. Mexico City: CEMEX. 501 p.
